# Parents’ and teachers’ perception about the impact of school nurse role in Saudi Arabia

**DOI:** 10.3389/fpubh.2025.1676311

**Published:** 2025-11-14

**Authors:** Eman Bajamal, Zainab AlShami, Ebtisam Aljahdali, Maha Alotaibi, Hanan Alharthi, Razan Alotaibi

**Affiliations:** 1 College of Nursing, King Saud Bin Abdulaziz University for Health Sciences, Jeddah, Saudi Arabia; 2 King Abdullah International Medical Research Center, Riyadh, Saudi Arabia

**Keywords:** school nurse, quantitative research, parents’ perception, teachers’ perception, Saudi Arabia

## Abstract

**Purpose:**

To assess the parent’s and teacher’s perception of the school nurse role in Saudi Arabia.

**Methods:**

A cross-sectional study of 305 participants of parents and teachers in Saudi Arabia. Data was collected using a previously tested and validated questionnaire to measure the parents and teacher perception about the role of school nurse.

**Results:**

Approximately 305 participants completed the online questionnaire. The total perception scores ranged from 0.00 to 34.00, with a mean of (*M* = 27.52, *SD* = 5.96). The scale demonstrated excellent internal consistency (Cronbach’s *α* = 0.902; McDonald’s *ω* = 0.896). Exploratory factor analysis supported a four-factor structure, explaining 58.6% of the total variance. The results revealed that the divorced participants had significantly lower perception of the school nurse’s role than married and widowed participants. Similarly, participants with lower family income demonstrated lower perceptions of the school nurse role than higher family income participants. Additionally, uneducated participants had significantly lower perceptions of the school nurse’s role in providing first aid and emergency care compared with high school education/undergraduate/graduate degree participants.

**Conclusion:**

The findings highlight that most participants perceived the school nurse as playing a vital role in delivering comprehensive health services within the school setting. This underscores the significance of strengthening school nursing services to support student well-being and health promotion.

## Introduction

School-aged children spend most of their day in school, the site for most of the learning and socialization. These years in children’s lives are characterized by crucial biological and social developments that mold their personality (1). The early educational period is mainly crucial because it comprises processes through which a child integrates habits, beliefs, and competencies necessary for the maturation of life in adults (2). This makes the time spent in school a significant determinant of lifelong health and well-being among children (1).

Recently, the school setting has appeared to be a basic area for promptly detecting and managing health problems in students (3). The complexity of students’ health problems dictates an increase in the role of school nurses because they are regarded as agents of the health system in schools. The school nurse’s role in this respect is not only to provide direct care but also to take part in the provision of different types of support to families and school personnel (2). School nursing is “a specialized public health nursing field that protects and promotes student health, enables normal development, and promotes academic success” (4, p. 84). The school nurse is a fundamental member of the school system workforce (5). He/she serve in effective roles in protecting, promoting, and evaluating the health of students and the school workforce (18). As integral members of the school health team, school nurses direct health-related initiatives and work with school staff, parents, and community members to ensure the health and safety of the school populace to support seamless learning (3).

Among the various roles of school nurses are providing preventive care and administering treatments for minor injuries and emergencies (Temel et al., 2019). They also make referrals and are crucial members of the healthcare team in managing chronic illnesses and preventing complications (6). School nurses develop and implement both long- and short-term health plans, which include services related to immunizations, health maintenance, and ensuring environmental safety and protection for all students (7). Additionally, school nurses work in interdisciplinary teams within the health sector to track cases of chronic illness and infectious diseases (8). They also provide education to teachers, school staff, guardians, and parents (7). School nurses have a unique responsibility and opportunity to work directly and actively with students to promote health and wellness (9).

## Background

Perception is the critical identification and interpretation of presented knowledge and experiences. It is an important element in increasing awareness and enhancing the quality of any given care (10). Teachers’ perceptions are crucial for understanding and supporting the roles of school nurses (11). The way teachers perceive the role of school nurses can significantly impact their level of cooperation in achieving the best health outcomes for students. Similarly, parents’ perceptions of school nurses’ roles can influence their involvement and collaboration with healthcare authorities, which is essential for overcoming barriers and facilitating preventive healthcare efforts in maintaining children’s health (7).

In addition to the existing body of quantitative studies, several qualitative and mixed-methods studies have also surveyed parents’ and teachers’ perceptions towards school nurses using interviews and in-depth analysis. For example, Mäenpää and Astedt-Kurki (12) conducted parent interviews to explore their perceptions on cooperation with school nurses in Finland, revealing gaps in communication and trust issues. Concurrently, Nguyen and Somrongthong (13) examined Vietnamese parents’ understanding and found both cultural expectations and avoidance of use of school health services. Clark et al. (14) measured parent understanding and attitudes within an urban US environment and recognized conflicts between parental and service delivery expectations. Baisch et al. (15) emphasized the role played by school nurses within an urban American school district and utilized evidence-based practice in advocating for more investment in school health infrastructure. Such studies offer strong contextual depth that complements the body of research and validate the necessity of exploring not just awareness, but also attitudes, communication behaviors, as well as systemic concerns. Incorporation of these findings strengthens the theoretical foundation of this study and underlines the need for context- and culture-oriented strategies in Saudi Arabia, where school health services remain to be fully established.

The literature indicates a significant misperception and underestimation of the school nurse’s role by teachers, highlighting a range of opinions and concerns. Maida et al. (16) found that teachers’ views on the school nurse’s role varied widely, from appreciation to negative criticism. Some teachers expressed concerns about the nurses’ availability and presence in schools, while others acknowledged the nurses’ capability to interact with parents and serve as a referral resource to community services. Similarly, McCluskey et al. (3) reported that teachers perceived the importance of the school nurse’s role as being closely linked to the nurses’ availability and visibility within the school setting. The perceived significance of the school nurse was often minimized due to the limited availability and inconsistent presence of nurses in schools (17). Additionally, some teachers viewed the school nurse’s role as restricted to providing first aid during emergencies (18). This limited view led to the underestimation and lack of recognition of the school nurse as a crucial solution for student health issues.

These findings are primarily founded on Western educational settings, in particular North America and Europe, where school health services have evolved in more established public health structures (19, 20). The Saudi Arabian education and health systems, however, demonstrate another model, driven by cultural influences, evolving healthcare policy, and organizational functions that are still emerging for integrated school health services. Saudi Arabia is in possession of a less institutionalized and newer school nurse role, which may help form differences in perception between teachers and parents. The school nurse role is less profiled and not in place at every level of regions or schools (21, 22). Therefore, as international studies recognize variation in perception due to availability and roles’ clarity, the Saudi Arabian context demands further scrutiny of how these roles are perceived, made easy, or overlooked in daily school life. Often, perceptions of the role among parents mirror those of the teacher, with many underestimating several components of the school nurse’s role. For example, parents view school nurses as mainly involved in providing first aid and medications, giving little value to their roles in communication and disease prevention. In addition, follow-ups, making referrals to providers, and administering medications are viewed as less valuable to parents (7, 18). This is similar to findings by Kirchofer et al. (23) with regard to parents, who perceived the role of the school nurse to be secondary to that of maintaining and improving student health. Many of them thought that the main work of the nurse was to handle emergencies. This narrow view limits the visibility and recognition of a wider scope of the school nurse’s role. The literature indicates that building positive perceptions among parents and teachers regarding the contribution of school nurses can substantially improve the nurse’s effectiveness in health promotion, disease prevention, and early detection of health problems (2). Understanding and higher appreciation of the role of school nurses could improve the health outcomes of students and health services and foster a more integrated approach to school health services.

In Saudi Arabia, school nurses play an essential role within the Healthy Schools Framework, a program that has been recommended for more than 20 years in the country. This program, under the title Health-Promoting Schools, includes different dimensions, such as school health services, health education for students and staff, relation with the surrounding community, food safety, physical activity, and mental health and counseling services (24). Still, a study that explored the initiation of health-promoting schools in Saudi Arabia reported that activities that address health education, as a concept, were most frequent, while activities that included the social environment and the community links, including the parents and the teachers, were the least initiated (25). Again, this should not be unexpected. Parents and teachers tend to underestimate the role that school nurses can play in health promotion and disease prevention because they have learned to recognize health specialists under other titles. On the other hand, an attempt can be made to change this view. More directly, parents and teachers should be forced to reconsider their stance by getting to know and appreciating the role that school nurses can play in their children’s health. This need is made even more pressuring by the fact that significant health-related problems have been located in Saudi school children, such as the recent increase in childhood obesity. More than 25% of Saudi school children are overweight or obese, proving that this is not a random but a pattern reflected through several years, seemingly due to the process of globalization that has infiltrated every aspect of modern life. Since this is the case, health problems like obesity or anorexia require multi-level solutions, from the creation of healthy home environments to the cultivation of proper school settings (26, 27).

Research on the perceptions of teachers and parents regarding school nursing is notably sparse, as highlighted in recent literature (9). In Saudi Arabia, this gap is even more pronounced. Understanding these perceptions is crucial for enhancing the role and effectiveness of school nurses.

The absence of available data on how teachers and parents perceive the role of school nurse underscores the importance of conducting this study. The findings from this study may give insight into the perceptions held by teachers and parents. Achieving complete comprehension about the perceptions of the role of the school nurse may give good opportunities in educating parents and teachers on the importance of school nurses and the multidimensional roles the school nurses have. Having positive perceptions of school nurses’ roles may help parents and teachers trust in the care their child receives while receiving their educations at school, and may these parents and teachers seek assistance from the school nurses. Therefore, the purpose of this study was to assess the parents’ and teachers’ perception about school nurse role in Saudi Arabia.

## Method

### Design and setting

A cross-sectional design was used for this proposed study, which is a non-experimental observational design that involves collecting data from participants at a single point in time. This design is particularly useful for assessing the current perceptions and attitudes of parents and teachers regarding the role of school nurses in Saudi Arabia. This study was conducted among parents and teachers in different regions of Saudi Arabia.

Participants with all of the following criteria were included in the study: (1) Parents who have children and adolescents in schools in Saudi Arabia; and (2) Teachers who directly teach or dealing with children/adolescents in schools in Saudi Arabia. Participants were excluded from the study if they are: (1) Parents who do not have children/adolescents in schools; and (2) Teachers who indirectly teach with children/adolescents in schools. All the participants were invited to participate in this study; however, the participation was voluntary and the sampling process were continued until the fulfillment of the sample size from the targeted population.

### Sample size

The initial sample size was estimated by G*power software, which allows sample size analysis and high-precision power and computes the power values for sample size, effect size, and alpha levels. The aim to include *n* = 176 to achieve the power of 95% with a medium effect size = 0.3, error probability = 5%, and a missing data is estimated as 10%. The total sample size is *N* = (176 + 18) = 194. Fortunately, the online questionnaire was completed by 305 participants, which is more than the initially estimated sample size. The larger sample size enhances the representativeness and significance of the study results.

### Sampling technique

The sampling technique that was used by the researchers is snowballing which is a non-probability sampling method where existing study participants recruit future participants from among their acquaintances. This process continues until the researcher reaches the desired sample size (28). The questionnaire was sent using different social media applications, such as WhatsApp, Telegram, Tweeter, Facebook, and emails.

### Data collection methods, instruments used and measurements

The data collected in this study using valid and reliable tool that have been validated and found to be reliable in several previous studies to measure the parents and teacher perception about the role of school nurse. The questionnaire included two parts. The first part included socio-demographic information of the participants, such as age, gender, nationality, marital status, education level, and monthly income. The second part included “Perceived Importance of School Nurse’s Roles in School Setting Questionnaire” that collects data related to parents’ and teachers’ perception of school nurse role. The questionnaire consists of 17 items. Each item is scaled as 0 if the response is “Not important,” (1) if the response “Important” and (2) if the response “very important.” The total score of the questionnaire is 34. Having a total mean score-less than 17 means that the participants perceive school nurse role as not important. Having a total mean score ranges from 17 to 24 means that the participants perceive school nurse role as important. Having a total mean more than 24 means that the participants perceive school nurse role as very important (23).

The 17 items of the questionnaire collect perceptions of the participants about the roles of school nurse. The roles included in the questionnaire are: (1) providing first aid and emergency care to students; (2) communicating with parents, school and health providers when a health problem is existing; (3) preventing and controlling diseases and creating a positive school environment by monitoring immunization and monitoring environmental safety; (4) educating teachers on the health requirements for students; (5) providing medical treatments to students; (6) teaching students about health issues; (7) working with school team on health, safety and educational issues; (8) performing health screening (vision, hearing, dental); (9) maintaining student health record (immunizations, screenings); (10) providing health education and services to the school staff; (11) identifying health problems; (12) giving prescription medication; (13) following up students who miss school; (14) giving over the counter medications; (15) providing referrals to local agencies and services; (16) conducting follow-ups on referrals to local agencies and services; and (17) helping in creating a positive school environment (23). Stability reliability was reported for the construct of the scale using Pearson product moment correlation coefficients (*r* = 0.77) (23).

### Scale translation and validation

In the current study, the scale was translated into Arabic using the forward-backward-forward translation technique, following the Principles of Good Practice for Translation and Cultural Adaptation (29). To ensure linguistic clarity and contextual appropriateness, a pilot test was conducted with a sample of 30 participants, including both parents and teachers.

### Data management and analysis plan

Once all the data was collected, the researcher encoded this data into an Excel database for analysis. The Statistical Package for the Social Sciences (SPSS for Mac, Version 21.0) was used to analyze the data. Descriptive statistics were computed for all desired variables, including the means, standard deviations, frequencies, and percentage, to describe study variables. One-way analysis of variance (ANOVA) and Tukey’s HSD (Honestly Significant Difference) *Post Hoc* tests were conducted in examining the group differences across demographic variables. Pearson’s correlation coefficients were employed to explore the associations among scale items and assess convergent validity. A significance level of 0.05 was set for all statistical tests. To assess the construct validity of the Arabic-translated “Perceived Importance of School Nurse’s Roles” scale, an Exploratory Factor Analysis (EFA) was conducted using Principal Axis Factoring with Varimax rotation. The Kaiser-Meyer-Olkin (KMO) measure and Bartlett’s Test of Sphericity were used to assess the adequacy of the data for factor analysis. Additionally, internal consistency was evaluated using Cronbach’s alpha and McDonald’s omega coefficients. These analyses collectively aimed to provide a comprehensive understanding of parents’ and teachers’ perceptions regarding the school nurse’s role in Saudi Arabian schools.

### Ethical consideration

Institutional Review Board (IRB) approval was obtained from research unit (CON-J) and King Abdullah International Medical Research Center (KAIMRC). Various measures were undertaken to protect the rights of study participants and to assure confidentiality. Participants were identified through code numbers rather than personal identifiers. Personal data collected were separated from documents containing identifying information, such as consent forms. The questionnaire was designed using a web-based questionnaire and it did not contain any section asking for named or ID of participants. Responses were kept anonymous to protect participant privacy. The consent was included as the first section of the online questionnaire. Participants must click accept to participate, which allows them to proceed to the questionnaire. The consent form was stated that the participation is completely voluntary and that participants have the right to withdraw from the study at any time without any consequences.

## Results

### Sample characteristics

A total of 305 participants completed the online questionnaire. As shown in Table 1, the largest percentage of the participants were parents (*n* = 236, 77.4%) compared to teachers (*n* = 56, 18.4%), while only (*n* = 13, 4.3%) were parents and teachers at the same time. Most participants were Saudi (93.8%) and female (90.8%). The participants’ ages ranged from 20 to over 60 years, with the largest age groups being 41–59 years (43.3%) and 31–40 years (41.3%). Additionally, the majority of participants were married (91.8%), while a small proportion were widowed (1.60%). A significant majority of participants hold an undergraduate degree (Bachelor/Diploma; *n* = 198, 64.9%), while the smallest proportion was uneducated (*n* = 5, 1.60%). The majority of participants reported a family’s monthly income of SR10,000 and above (*n* = 94, 30.8%), while the lowest income represented was SR3001-SR5000 (*n* = 55, 18.0%).

**Table 1:** Sample demographic characteristics (*N* = 305).

Demographic variable	*n*	%
Participant status		
Parent	236	77.4
Teacher	56	18.4
Parent and teacher	13	4.30
Nationality		
Saudi	286	93.8
Non-Saudi	19	6.20
Age group (years)		
20–30	41	13.4
31–40	126	41.3
41–59	132	43.3
60 and above	6	2.00
Gender		
Male	28	9.20
Female	276	90.8
Marital status		
Married	279	91.8
Divorced	20	6.60
Widowed	5	1.60
Education level		
Uneducated	5	1.60
High School	73	24.0
Undergraduate degree (bachelor/diploma)	197	64.8
Graduate degree (master/PhD)	29	9.50
Family monthly income (SR)*		
< 3,000	81	26.6
3,001–5,000	54	18.0
5,001–10,000	75	24.7
> 10,000	94	30.9

*USD $1 = SR 3.75.

Among 302 participants, only 15.1% reported that their child’s school had a school nurse, while the majority (83.9%) indicated the absence of the school nurse. Over one-third of participants (35.1%) reported that their child had health issues, with diabetes (9.20%), asthma (6.6%) and allergies (5.20%) being the most commonly reported conditions.

To compare perceptions between parents and teachers, participants who identified as both (*n* = 13) were excluded from subgroup analysis to ensure clear group distinctions. This resulted in a total of 286 participants (230 parents and 56 teachers) included in the comparative analyses.

### Perception of school nurse role

As presented in Table 2, the total perception scores ranged from 0.00 to 34.00 (*M* = 27.52, *SD* = 5.96). Out of 305 participants, 298 participants provided valid response regarding their perception of the school nurse’s role. The majority of respondents (*n* = 210, 70.5%) perceived the school nurse’s role as very important, while 26.2% (*n* = 78) viewed it as important. Only 3.40% (*n* = 10) perceived the role as not important. These results indicate a generally high appreciation of the school nurse’s role among respondents. Twelve responses (3.9%) were missing and not included in the analysis.

**Table 2:** Descriptive statistics of questionnaire items (*N* = 305).

Perception level	Frequency (*n*)	Valid percent (%)
Not Important	10	3.4
Important	78	26.2
Very Important	210	70.5
Total (valid)	298	100.0

Perception categories were based on the total score of the 17-item scale: <17 = not important, 17–24 = important, >24 = very important. Twelve cases were excluded due to missing data.

### Perceived importance of specific roles

As shown in Table 3, the descriptive statistics show that participants generally perceived all roles of school nurses as highly important. The highest-rated item was “Communicates with parents, school, and health providers” (*M* = 1.84, *SD* = 0.40), followed closely by “Provides first aid and emergency care to students” (*M* = 1.83, *SD* = 0.41), and “Helps create a positive school environment” (*M* = 1.73, *SD* = 0.49). The lowest-rated roles, though still seen as important, were “Gives prescription medications” (*M* = 1.35, *SD* = 0.71), “Gives over-the-counter medications” (*M* = 1.38, *SD* = 0.69), and “Follows up on referrals to local agencies” (*M* = 1.44, *SD* = 0.64), Overall, the low standard deviations across most items suggest consistency in participant responses.

**Table 3:** Descriptive statistics for perceived importance of school nurses roles (*N* = 305).

Item no.	School nurse role item	*N*	*M*	*SD*
1	Provides first aid and emergency care to students	304	1.83	0.41
2	Communicates with parents, school, and health providers	304	1.84	0.40
3	Prevents and controls diseases and monitors safety	304	1.71	0.50
4	Educates teachers on student health requirements	304	1.72	0.47
5	Provides medical treatments to students	304	1.64	0.58
6	Teaches students about health issues	304	1.67	0.54
7	Works with school team on health, safety, and education	304	1.66	0.53
8	Performs health screening (vision, hearing, dental)	303	1.64	0.57
9	Maintains student health records	304	1.64	0.58
10	Provides health education and services to staff	303	1.71	0.48
11	Identifies health problems	303	1.64	0.55
12	Gives prescription medications	304	1.35	0.71
13	Follows up on students who miss school	304	1.47	0.70
14	Gives over-the-counter medications	304	1.38	0.69
15	Provides referrals to local agencies and services	303	1.46	0.63
16	Follows up on referrals to local agencies	304	1.44	0.64
17	Helps create a positive school environment	302	1.73	0.49

Response options ranged from 0 = Not important to 2 = Very important.

### Comparison between parents and teachers

Independent samples t-tests were conducted to explore differences in total perception scores and individual item ratings between parents and teachers. Overall, parents (*M* = 27.49, *SD* = 6.00) and teachers (*M* = 27.71, *SD* = 5.89) demonstrated similarly high perceptions of the school nurse’s role, with no statistically significant difference, *t* (283) = − 0.25, *p* = 0.803. Additionally, independent t-tests on each of the 17 individual items revealed no significant differences between the two groups (*p* > 0.05 for all items). Both groups rated communication, emergency care, and promoting a positive school environment as highly important. A chi-square test comparing categorical perception levels (not important, important, very important) also showed no significant differences between groups, χ^2^ (2, *N* = 285) = 1.08, *p* = 0.584. Most respondents in both groups rated the school nurse’s role as very important.

### Scale reliability and validity

The reliability of the 17-item scale was assessed to ensure the internal consistency. The results demonstrated excellent reliability, with a Cronbach’s alpha (*α* = 0.902) and the McDonald’s Omega coefficient (*ω* = 0.896), both exceeding the conventional threshold of 0.80, indicating strong internal consistency across the scale items.

Additionally, the convergent validity was examined through inter-item Pearson correlations coefficients. As shown in Table 5, all items were significantly and positively correlated, with coefficients ranging from *r* = 0.118 to *r* = 0.645 (*p* < 0.05 or *p* < 0.01). These results indicate moderate to strong positive relationships between items (34), suggesting that the scale items consistently measure related dimensions of the same underlying construct for perceptions of the school nurse’s roles.

**Table 5:** Pearson’s correlation between tool items (*N* = 305).

Item no.	1	2	3	4	5	6	7	8	9	10	11	12	13	14	15	16	17
1	-																
2	0.624**	-															
3	0.477**	0.562**	-														
4	0.550**	0.517**	0.548**	-													
5	0.403**	0.463**	0.468**	0.512**	-												
6	0.260**	0.350**	0.311**	0.424**	0.420**	-											
7	0.275**	0.351**	0.411**	0.412**	0.355**	0.591**	-										
8	0.277**	0.305**	0.282**	0.365**	0.339**	0.506**	0.601**	-									
9	0.244**	0.285**	0.311**	0.407**	0.392**	0.581**	0.595**	0.645**	-								
10	0.395**	0.446**	0.412**	0.542**	0.452**	0.442**	0.375**	0.378**	0.467**	-							
11	0.404**	0.489**	0.401**	0.469**	0.467**	0.396**	0.396**	0.494**	0.412**	0.570**	-						
12	0.225**	0.239**	0.211**	0.339**	0.520**	0.406**	0.322**	0.344**	0.387**	0.389**	0.557**	-					
13	0.228**	0.312**	0.361**	0.442**	0.385**	0.348**	0.345**	0.335**	0.407**	0.504**	0.467**	0.586**	-				
14	0.160**	0.124*	0.153**	0.258**	0.244**	0.308**	0.276**	0.279**	0.308**	0.297**	0.234**	0.221**	0.152**	-			
15	0.173**	0.185**	0.137*	0.305**	0.287**	0.407**	0.354**	0.295**	0.363**	0.318**	0.302**	0.211**	0.161**	0.636**	-		
16	0.118*	0.155**	0.099	0.240**	0.271**	0.346**	0.312**	0.255**	0.322**	0.329**	0.313**	0.243**	0.198**	0.615**	0.848**	-	
17	0.239**	0.267**	0.184**	0.268**	0.189**	0.282**	0.371**	0.292**	0.370**	0.263**	0.289**	0.161**	0.170**	0.474**	0.488**	0.484**	-

*p < 0.05, **p < 0.01.

To assess construct validity, the Exploratory Factor Analysis (EFA) was conducted using Principal Axis Factoring with Varimax rotation. The Kaiser–Meyer–Olkin (KMO) measure of sampling adequacy was 0.885, indicating that the sample was well-suited for factor analysis. Bartlett’s Test of Sphericity was statistically significant (χ^2^ (136) = 2649.74, *p* < 0.001), suggesting sufficient correlations among items for EFA. The analysis yielded four distinct factors, which together explained 58.6% of the total variance. All factor loadings exceeded the minimum threshold of 0.45, and most item communalities ranged between 0.47 and 0.83, supporting the suitability of the items for inclusion within each factor (see Table 4). The four interpretable factors were as follows:

**Table 4:** EFA with Varimax rotation for the 17-Item school nurse role perception scale (*N* = 305).

Item no.	School nurse role item	Communality (extraction)	Factor 1	Factor 2	Factor 3	Factor 4
1	Provides first aid and emergency care to students	0.549	0.722			
2	Communicates with parents, school, and health providers	0.655	0.778			
3	Prevents and controls diseases and monitors safety	0.504	0.656			
4	Educates teachers on student health requirements	0.57	0.63			
5	Provides medical treatments to students	0.473	0.455			0.439
6	Teaches students about health issues	0.538			0.586	
7	Works with school team on health, safety, and education	0.626			0.691	
8	Performs health screening (vision, hearing, dental)	0.58			0.681	
9	Maintains student health records	0.66			0.703	
10	Provides health education and services to staff	0.501			0.425	
11	Identifies health problems	0.545				0.506
12	Gives prescription medications	0.718				0.808
13	Follows up on students who miss school	0.516				0.634
14	Gives over-the-counter medications	0.493		0.668		
15	Provides referrals to local agencies and services	0.832		0.885		
16	Follows up on referrals to local agencies	0.827		0.886		
17	Helps create a positive school environment	0.378		0.517		

Extraction method: Principal Axis Factoring. Rotation method: Varimax with Kaiser normalization. Only factor loadings ≥ 0.40 are reported. Kaiser-Meyer-Olkin (KMO) = 0.885; Bartlett’s Test of Sphericity: χ^2^(136) = 2649.74, *p* < 0.001. The total variance explained = 58.60%.

- Factor 1: Communication and Care: Included items related to first aid, emergency care, health communication with parents and providers, and health education for staff. This factor explained the largest portion of variance and reflects core caregiving and coordination functions of school nurses.
- Factor 2: Referral and Follow-Up Services: Captured roles involving referrals to external health agencies and follow-up on those referrals, as well as broader community health integration efforts.
- Factor 3: Health Education and Records: Encompassed responsibilities such as maintaining student health records, health screenings, teamwork with school staff, and teaching health-related content to students.
- Factor 4: Medication and Attendance: Included prescription and over-the-counter medication administration, as well as monitoring attendance and identifying health-related absenteeism patterns.

These findings confirm the multidimensional nature of the school nurse’s perceived role and suggest that the instrument validly captures key role domains relevant to school health services.

As shown in Table 5, there was a strong and statistically significant positive correlation between conducting follow-up on referrals to local agencies and providing such referrals (*r* = 0.848, *p* < 0.01), indicating that these roles are perceived as closely linked. Similarly, working with the school team on health, safety, and educational issues was strongly associated with teaching students about health topics (*r* = 0.591, *p* < 0.01), and also with giving over-the-counter medications (*r* = 0.276, *p* < 0.01), suggesting a perceived interconnection between collaborative practices and direct student health services. Moreover, moderate positive correlations were found between maintaining student health records and both health screening (*r* = 0.645, *p* < 0.01) and teaching students about health issues (*r* = 0.581, *p* < 0.01), underscoring the integrative role of documentation in broader health education activities. Prescription medication administration was also moderately correlated with identifying health problems (*r* = 0.557, *p* < 0.01), indicating its perceived clinical relevance.

Conversely, weak but statistically significant positive correlations were observed between administering over-the-counter medications and communicating with parents, schools, and health providers (*r* = 0.124, *p* < 0.05), as well as between conducting follow-ups and providing first aid (*r* = 0.118, *p* < 0.05). These findings suggest these tasks may be perceived as more distinct components of the school nurse’s role. Overall, most inter-item correlations were statistically significant at the 0.01 level, supporting the convergent validity of the scale and indicating that the items collectively measure a coherent construct related to perceptions of the school nurse’s role.

### The relationships between sociodemographic variables and scale items

#### Marital status

The One-way analysis of variance (ANOVA) revealed significant differences in perceptions of school nurse roles based on marital status. For item 2 (Communicates with parents, school and health providers if health problem exists), there was a significant effect *F* (2, 304) = 6.10, *p* = 0.003. Divorced participants reported lower agreement (*M* = 1.55, *SD* = 0.61) compared to married (*M* = 1.85, *SD* = 0.37), and widowed participants (*M* = 2.00, *SD* = 0.000). Similarly, item 3 (prevents and controls diseases…) showed a significant effect, *F* (2, 304) = 5.93, *p* = 0.003, with divorced participants again rated lower (*M* = 1.35, *SD* = 0.81) than married (*M* = 1.74, *SD* = 0.46), and widowed (*M* = 1.80, *SD* = 0.45). For item 5 (provides medical treatments), divorced participants (*M* = 1.15, *SD* = 0.81) rated lower than widowed (*M* = 1.60, *SD* = 0.55) and married (*M* = 1.68, *SD* = 0.54), *F* (2, 304) = 8.29, *p* < 0.001. No other items showed significant differences by marital status.

#### Monthly income

Participants’ perceptions differed significantly based on monthly income. For item 1 (provides first aid and emergency care to students), *F* (3, 301) = 4.18, *p* = 0.006, participants earning SR3001-SR5000 reported lower scores (*M* = 1.73, *SD* = 0.45) than those earning SR5001- SR10,000 (*M* = 1.91, *SD* = 0.29) or above SR10,000 (*M* = 1.90, *SD* = 0.36). For item 2 (Communication with parents, providers) also showed significant variation, *F* (3, 301) = 2.91, *p* = 0.035. The participants with income <SR3000 had lower perception (*M* = 1.74, *SD* = 0.45) compared to those earning > 10,000 (*M* = 1.91, *SD* = 0.28). For item 4 (Educates teachers) showed a trend toward significance, *F* (3, 301) = 2.29, *p* = 0.079, with lower means in the lowest income group. No other items showed significant differences.

#### Educational status

A one-way ANOVA revealed significant differences across educational groups. For item 1, *F* (3, 301) = 4.12, *p* = 0.007, uneducated participants reported lower agreement (*M* = 1.40, *SD* = 0.55) than those with graduate degrees than those with graduate degrees (*M* = 1.97, *SD* = 0.19). For item 4, also showed significant effect, *F* (3, 301) = 3.60, *p* = 0.014, with uneducated participants scoring lowest (*M* = 1.20, *SD* = 0.45). Other items did not show statistically significant differences by education level.

## Discussion

School nurses have a crucial part to play in attending to the health and well-being of students within the school. It is important for parents and teachers to know the roles of school nurses and maintain a good level of communication with these school nurses so that quality care services are provided for the benefit of students. In this context, it is an encouraging finding, in fact, that the parents and teachers in the study by McCluskey et al. (3) showed relatively good knowledge about the roles of school nurses. This kind of knowledge and collaboration among the stakeholders can contribute significantly toward promotion in schools.

The consistency in findings across various studies, including those Al Kindi et al. (7), Temel et al. (18), and Gross et al. (30), underscores the importance placed by parents and teachers on the primary role of school nurses in providing first aid and emergency care to students. This role is crucial in ensuring the immediate health and safety of students and involves effective communication with parents, school authorities, and healthcare providers in case of health concerns. This was further confirmed by the current study, in which “Provides first aid and emergency care” and “Communicates with parents, school, and health providers” received the highest mean ratings. Moreover, these two items were also positively correlated (*r* = 0.624, *p* < 0.01), reinforcing their interconnected perception as essential roles.

On the other hand, the study by Martinez et al. (2) presented contrasting results, indicating that participants with formal authority or influence in healthcare settings viewed the provision of first aid and emergency care as less important compared to roles like wound treatment, sutures, and bandages. This discrepancy highlights the diverse perspectives on the roles of school nurses, which may vary based on the stakeholders’ backgrounds and experiences within the healthcare system.

The alignment of findings from Pestaner et al. (11), Hilli and Pedersen (8), Heggestad et al. (31), and Maughan et al. (33) with the current study regarding the importance of school nurses’ role in communicating with parents, schools, and health providers when health issues arise is noteworthy. These studies consistently highlighted that parents and teachers perceive this communication aspect as one of the most important roles of school nurses. This was supported in the present study, with this role receiving the highest mean score (*M* = 1.84, *SD* = 0.40). It was also strongly correlated with related clinical roles, such as providing first aid (*r* = 0.624), identifying health problems (*r* = 0.489), and providing referrals (*r* = 0.185), all at significant levels. Effective communication in such situations is essential for ensuring coordinated care and support for students facing health challenges. In contrast, the study by Martinez et al. (2) presented a different perspective, ranking the role of communicating with parents, schools, and health providers lower in importance compared to other roles. This discrepancy further emphasizes the variability in perceptions regarding the roles and priorities of school nurses, which may be influenced by the context, backgrounds, and experiences of the individuals involved in the studies.

The discrepancy in the ranking of the role of maintaining student health records, including immunizations and screenings, among the studies by Kirchofer et al. (23) and Maughan et al. (2011), and the current study is an interesting observation. While Maughan et al. (2011) and Kirchofer et al. (23) identified this role as being among the fifth most important responsibilities of school nurses according to parents and teachers, the current study ranked it as ninth in importance (*M* = 1.64, *SD* = 0.58). Despite the relatively lower ranking, its strong correlation with health screening (*r* = 0.645) and health education to students (*r* = 0.581) underscores its indirect value in supporting broader school health objectives. This difference in ranking suggests that perceptions of the significance of maintaining student health records may vary among different study populations or contexts.

It is interesting to note the consistency in findings across multiple studies, including those by Kirchofer et al. (23) and Maughan et al. (2011), regarding the perceived importance of various roles of school nurses as reported by parents and teachers. The roles of preventing and controlling diseases, creating a healthy school environment, checking for immunizations and safety concerns in the school environment, and educating teachers on health requirements and medical treatments for students were among the top responsibilities of school nurses emerging in both studies. These roles were also reflected in the current findings. For example, “Works with the school team on health, safety, and education” was strongly correlated with “Teaches students about health issues” (*r* = 0.591, *p* < 0.01) and “Gives over-the-counter medications” (r = 0.276, *p* < 0.01), illustrating how interprofessional teamwork supports multiple functions.

It is interesting to consider the similarities and differences in results from the current study, with Kirchofer et al. (23) and Martinez et al. (2) in terms of the perceived importance of some roles of the school nurse to parents and teachers. Consistent with Kirchofer et al. (23) and Martinez et al. (2), the least important role of the school nurse according to parents and teachers in the current study was giving prescription medication (*M* = 1.35, *SD* = 0.71) and following up with missed students. Despite this lower ranking, these roles still showed moderate associations with other functions; for instance, giving prescription medication was moderately correlated with identifying health problems (*r* = 0.557, *p* < 0.01), highlighting their clinical relevance. However, no similar findings were found from the data that would indicate that either demographic variable could have an impact on the roles that the stakeholders perceived the school nurse should be involved in. This stands in contrast with the findings by Drakopoulou et al. (17) and Kirchofer et al. (23) in which the results did show that associations were present based on demographic variables. However, the characteristics of the differences in the existence of the correlations, based on demographic variables, point out the nuanced nature of how a variety of factors will impact how the stakeholders will view the roles and responsibilities of the school nurse across various study populations.

### Limitations

The participants of this research sample were recruited via snowball sampling, which is a referral technique. One or more participants are initially contacted, after which the next wave of participants are referred, creating a chain. This technique ensures that the participants have similar characteristics or know each other, but it does not ensure that every individual in the population has an equal opportunity to be included. Therefore, sampling bias may result. Besides, the study design is cross-sectional. It relies on the willingness of individuals to respond to questionnaires, which may reflect non-response bias. When population segments are more willing to participate, an over-representation is created, potentially making the study’s findings less reliable (32).

### Recommendations

As a school nurse is an important part of raising awareness about their manifold roles, and informing parents, teachers, and the community is necessary for the full utilization of their services (8, 11, 17, 33). School nurses are key partners with families; thus, parents should be informed about school nurses’ multiple roles. If parents and teachers are not aware of this role, they may not be able to make the best use of this resource. Informing parents and teachers through holding educational sessions for parents and teachers within the school locations would expand knowledge about what school nurses do and promote cooperation for the best outcomes for children’s health. School nurses, with the help of the principals, must ensure that parents are aware of all the functions of the school nurse; how to access the school nurse services, the importance of communicating any health concern, and maintaining consistent communication. The school nurses can also hold annual educational sessions, or meetings for parents and teachers to explain their role and offer assistance. It can be during this time that they can offer response to any needs or questions from parents or teachers. For future research studies, it is recommended to employ a sampling technique other than snowball sampling to avoid biases from participants with similar characteristics or who know each other. This would improve the reliability of the results. Second, it is recommended that future researchers employ strategies or ways of addressing non-response biases that are associated with survey or questionnaire designs in cross-sectional designs.

### Implications for future practice

Several essential measures need to be in place to create a better impression and elevate the school nurses’ status in the eyes of teachers and parents. Education sessions is one of the keys by conducting workshops and informative sessions for parents and teachers regularly. The session will describe the role of the school nurse in terms of health education, emergency care, chronic disease management, and mental health support among other comprehensive roles and responsibilities. Communication strategies through a clear outline of communication channels school nurses, parents, and teachers. This could be in terms of a newsletter, email or even a special page on the school’s website that outlines the school nurse’s activities and how to reach her. Regular updates about health-related school policies and student health programs and any change in the role or the responsibility of the school nurse.

Moreover, school administration collaboration that involves school principals in actively supporting and promoting the role of the school nurse. Principals must involve nurses in meetings and other school decisions concerned with the health of the children. Parental involvement through developing health education programs created specifically for parents. This increases their level of knowledge regarding child health and the essential role played by school nurses in child health. Create a mechanism for parental feedback, such that parents can express concerns and make suggestions on the health services from the school. This helps to better shape the services to meet the needs of the student. Moreover, enhancing the visibility of the school nurses within the school setting. This could involve attending parent-teacher meetings, school events, and being visible to the student throughout the school hours. Cross-training, where school nurses can encourage cross-training sessions to train the teachers and school staff on basic health practices and emergency response. Furthermore, supporting implementation of evidence-based practices aimed at improving student health results and explaining successes with these programs to parents and teachers builds trust and appreciation for the role of school nurses. These are ways by which school nurses can better the parents’ and teachers’ perceived role toward supporting improvement of health outcomes among students and a more conducive environment for learning.

## Data Availability

The raw data supporting the conclusions of this article will be made available by the authors, without undue reservation.
